# Cross-sex hormone therapy in rats induces sex-specific adaptations of renal function and sodium transporters expression

**DOI:** 10.3389/fphys.2025.1653915

**Published:** 2025-08-15

**Authors:** Debora Conte Kimura Lichtenecker, Nathalia Beserra da Silva, Isabela Borges Silveira, Letícia Maria Monteiro, Rogerio Argeri, Magnus R. Dias Da Silva, Guiomar Nascimento Gomes

**Affiliations:** ^1^ Department of Physiology, Escola Paulista de Medicina, Federal University of São Paulo, São Paulo, Brazil; ^2^ Postgraduate Program in Translational Medicine, Department of Medicine, Escola Paulista de Medicina, Federal University of São Paulo, São Paulo, Brazil; ^3^ Laboratory of Molecular and Translational Endocrinology (LEMT), Endocrinology Division, Department of Medicine, Escola Paulista de Medicina, Universidade Federal de São Paulo (EPM/Unifesp), São Paulo, Brazil; ^4^ Trans Care Outpatient Clinics, Núcleo de Estudos, Pesquisa, Extensão e Assistência à Pessoa Trans Professor Roberto Farina, Universidade Federal de São Paulo (Núcleo TransUnifesp), São Paulo, Brazil

**Keywords:** cross-sex hormone therapy, renal function, sodium transporters, glomerular function, blood pressure

## Abstract

**Introduction:**

Cross-sex hormone therapy (CHT) has been used in the gender identity-affirming process. Nevertheless, the literature about the renal repercussions of this therapy is scarce.

**Objective:**

Evaluate the effects of CHT on blood pressure (BP) and renal function.

**Methods:**

Male and female Wistar rats were distributed into groups: M + H (male + hormone), M + V (male + vehicle), F + H (female + hormone), and F + V (female + vehicle). CHT: M + H received algestone-acetophenide (3 mg/kg) plus estradiol-enanthate (0.18 mg/kg); F + H, testosterone-cypionate (3 mg/kg). The vehicle was sesame oil. After 2 months of treatment, BP and renal function [inulin clearance (GFR), ions, and acid excretions] were evaluated. Sodium transporters expression (NHE3, NCC, α-ENaC, and β-ENaC) was assessed by immunohistochemistry.

**Results:**

Compared to M + V, M + H presented reduction in BP and GFR but increase in sodium and potassium excretion. GFR did not change in F + H, but sodium and potassium excretions were reduced. Ammonium excretion was decreased in M + H but increased in F + H. The NHE3 expression decreased in M + H and increased in F + H; females showed higher expression of NCC, while CHT did not change it. The β-EnaC expression was higher in females; CHT increased it in males and females.

**Conclusion:**

CHT induces sex-specific renal adaptations. Testosterone in females reduces the excretion of sodium and other ions, which may predispose to hypertension. Conversely, estradiol + algestone in males decrease the glomerular filtration rate and alter sodium handling, suggesting maladaptive responses. The expression of sodium transporters was altered in a sex- and nephron segment-specific manner. These findings highlight the need for further studies on the renal consequences of hormone therapy in transgender individuals.

## Introduction

The use of cross-sex hormone therapy (CHT) has increased in recent years, mainly due to its beneficial role in supporting gender affirmation. This therapy involves the administration of steroid hormones, usually different from the predominantly produced endogenously. Testosterone is generally prescribed for transgender men to shrink the levels of estradiol and other female-associated hormones, promoting the development of male secondary sexual characteristics (Figueiredo et al., 2022; Irwig, 2017). In contrast, trans-women typically receive combinations of estradiol and other agents aimed at suppressing testosterone production and facilitating feminization (Castilla-Peón, 2018; Collister et al., 2021). Despite its widespread use, the physiological repercussions of CHT are still not well defined.

Sex-related differences in the morphological and functional aspects of several organs have been linked to the development of various diseases, including kidney disorders (Lindsey et al., 2023). Regarding renal physiology, experimental studies in rodents have suggested sex-specific structural variations in the renal tubules, as well as distinct patterns in the distribution of sodium transporters (Harris et al., 2018; Veiras et al., 2017). These differences result in a major percentage of reabsorption in the proximal segments of males due to their increased reabsorption area and density of transporters. In contrast, females exhibit a higher density of sodium transporters in the distal nephron segments, which play a key role in regulating sodium excretion and maintaining electrolyte balance (Hu et al., 2019; McDonough et al., 2024; Veiras et al., 2017).

Most of the studies about sex hormones, whether experimental or in humans, were conducted in cisgender individuals, and few experimental studies have evaluated the repercussions of CHT on conditions similar to those of transgender people (Dela Cruz et al., 2023; Gusmão-Silva et al., 2022; Kothmann et al., 2021; Lichtenecker et al., 2021; Tassinari and Maranghi, 2021). However, the impact of these therapies on the individual’s health is a topic of public interest.

Initial studies conducted in our laboratory using an experimental model with Wistar rats (Gusmão-Silva et al., 2022; Lichtenecker et al., 2021) indicated that CHT induces significant renal morphological changes, which vary according to sex and the therapeutic regimen employed. Given the need for a more comprehensive assessment of renal function to determine the specific adaptations triggered by CHT—particularly concerning renal hemodynamics and ion transport—this study aimed to evaluate the effects of CHT in male and female rats, focusing on blood pressure (BP) and renal morphofunctional parameters, including the expression of sodium transporters along different segments of the nephron.

## Methods

The animals used in this study were purchased from the Center for Development of Experimental Models for Medicine and Biology (CEDEME/UNIFESP). The two-month-old male Wistar rats weighed an average of 270 g and females 195 g. The animals were housed in ventilated cages and had free access to standard laboratory chow (Purina) and water throughout the experimental protocol in a room maintained at a constant temperature (22 °C) and with a 12-h light cycle (lights on at 7 a.m.). The experimental protocol was approved by the Research Ethics Committee of UNIFESP (CEUA: 9009241022) and adhered to international guidelines for the care of research animals.

The animals were distributed into four groups: (1) Male treated with vehicle, M + V; (2) Male treated with hormone, M + H; (3) Female treated with vehicle, F + V; and (4) Female treated with hormone, F + H. Hormonal treatment refers to cross-sex hormone therapy. Thus, the F + H group received testosterone cypionate (3.0 mg/kg, i.m.), while the M + H group received a combination containing algestone acetophenide (3 mg/kg, i.m.) and estradiol enanthate (0.18 mg/kg, i.m.). The F + V and M + V groups received vehicle (sesame oil). The hormone doses were calculated from the studies by Lichtenecker et al. (2021) and Gusmão-Silva et al. (2022). Intramuscular administration of hormone and vehicle occurred every 10 days for the 8 weeks of the experimental protocol. Each group had between 7 and 11 animals. This was determined based on statistical power calculations, previous studies and ethical considerations.

### Blood pressure measurement (BP)

Caudal blood pressure was measured at the end of treatment using the indirect tail plethysmography technique, as previously described (Argeri et al., 2022). From 3 months on, the animals were adapted to the pressure measurement system (Coda Monitor–Kent Scientific Corp. Torrington-CT, United States) through simulated procedures for 2 weeks before the procedure began. To measure, the animal was placed in an acrylic restraint cylinder, appropriate for its age, in a heating chamber at a constant temperature of 34 °C, to promote slight dilation of the caudal artery. A sphygmomanometer with a sensor, connected to a recording system was adjusted to the proximal portion of the rat’s tail (caudal artery). The cuff was inflated to 220 mmHg and then deflated, allowing the equipment to measure the systolic pressure. In each rat, four to five measurements were taken in sequence, and the caudal pressure value in mmHg was obtained by averaging these measurements (Argeri et al., 2022).

### Assessment of renal function—clearance assessment

Before clearance assessments, rats were placed in metabolic cages (Criffa, Barcelona, Spain) for 24 h. The collected urine samples were used to measure ion excretion. After the period in the metabolic cages, arterial blood samples were collected to determine pH, HCO3, pCO2, and pO2 on a clinical analyzer (i-STAT - Abbott Point of Care Inc.) and to determine the concentration of sodium, potassium, calcium, and magnesium ions on a Hitachi Cobas c702 analyzer (Roche Diagnostics, Indianapolis, IN, United States).

To assess clearance, we anesthetized the rats with sodium thiopental (Cristália) at a dose of 60 mg/kg i.p., and additional doses were administered during the experiment if the anesthetic plane of the animals became superficial.

Initially, we placed a polyethylene tube (PE 260) in the trachea of each animal to facilitate ventilation. Then, we inserted a polyethylene PE 20 cannula into the right carotid artery for blood collection. In the left external jugular vein, we placed a polyethylene PE 50 cannula for infusion of different solutions. We inserted a catheter (polyethylene PE 260) into the bladder for urine sample collection. After catheterization, we initiated a continuous intravenous infusion of 0.9% NaCl and 3% mannitol using an infusion pump (Harvard PHD 2000) at a rate of 100 μL/min for 30 min. We then administered an initial dose of inulin (Sigma) of 300 mg/kg of body weight, followed by a maintenance dose of 5 mg/min per kg of body weight. At the same time, we administered an initial dose of para-amino hippurate (PAH - Sigma) of 6.66 mg/kg of body weight (dissolved together with the initial dose of inulin) and a maintenance dose of PAH of 1.33 mg/min per kg of body weight. After 30 minutes of infusion (of the solutions containing inulin and PAH), we began the urine and blood collection periods for clearance determinations.

We calculated the glomerular filtration rate (GFR) and renal plasma flow (RPF) from the clearance of the respective substances (inulin and PAH). The blood samples were collected with heparinized syringes (Liquemine, Roche) and centrifuged at 5,000 rpm for 10 min (Fanem centrifuge, SP. B-204-NR). We separated the plasma and stored it in a refrigerator until the assays were done. We collected the urine samples under mineral oil in previously weighed glass tubes. After collection, we weighed the tubes again to assess the urine volume. The urine samples were stored under mineral oil in a refrigerator until the assays. We used colorimetric methods to determine the plasma and urine concentrations of inulin and PAH (Argeri et al., 2022).

### Morphological analysis

The kidneys were weighed and then fixed in Bouin’s solution and embedded in paraffin for morphological assessment. Histological sections (5 μm thick) were stained with hematoxylin and eosin. Images were acquired (200× magnification) on a microscope (Nikon H550L) connected to a microcomputer via a video camera (Sony CCD-IRIS) using the Nikon NIS-Elements software. The glomerular areas were measured in histological images obtained from twenty consecutive fields of the renal cortex per slide per animal. Each field presented around 2 to 3 glomeruli. The images were analyzed using ImageJ software (Schneider et al., 2012). For each identified glomerulus, the area was determined using the Freehand Selection tool, with manual contouring of the glomerular structures. Measurements were expressed in square micrometers (µm^2^), based on the calibrated scale of each image.

For the analysis of the expression of sodium transporters we performed the following immunohistochemical protocol: kidney slices fixed in slides were incubated overnight at 4 °C with anti-sodium transporters: sodium-hydrogen exchanger 3 (NHE3), 1:300, sodium-potassium-chloride co-transporter 2 (NKCC2), 1:300, sodium-chloride co-transporter (NCC), 1:300, and alpha or beta epithelial sodium channel (αENaC/βENaC), 1:300. All the primary antibodies used were acquired from StressMarq Biosciences Inc., Victoria, BO, Canada). The reaction products were determined using a universal immuno-peroxidase polymer (Histofine-Nichirei Biosciences). For quantitative analysis, an area of 20 consecutive cortical fields was selected for each sample (×200 magnification). Protein expression was quantified as the percentage of positively stained area in the tubulointerstitial compartment. Images were converted to grayscale and analyzed in ImageJ (Schneider et al., 2012). An intensity threshold was applied for segmentation of the positive staining, defined manually for each image based on the visual distinction between the specific signal and the background, in order to exclude unstained areas. The analysis was restricted to the tubulointerstitial region by manually selecting the area of interest with the Freehand Selection tool. The expression was then calculated as the ratio between the area of positive pixels (above the threshold) and the total selected area of the tubulointerstitium. Glomeruli and large vessels were carefully excluded from the selection.

### Statistical analysis

Results are presented as mean ± standard error and were analyzed by two-way ANOVA. Additionally, Tukey’s *post hoc* test was used for multiple comparisons between groups (Prism 6.0, GraphPad). Values of p ≤ 0.05 were considered significant changes.

## Results

Cross-hormone therapy caused metabolic adaptations that significantly modified the animals' weight gain. By the end of the experimental protocol the M + H group showed a significant reduction while the F + H group showed increase (Weight gain: M + V: 45.5 + 3.47; M + H: 7.0 + 3.25*; F + V: 22.5 + 1.46; F + H: 40.9 + 2.04*; % of initial bodyweight).

Another parameter that was significantly altered was the relative weight of the gonads, which was greatly reduced in both groups under hormonal treatment (Gonads' weight: M + V: 0.94 + 0.05; M + H: 0.33 + 0.05*; F + V: 0.43 + 0.02 and F + H: 0.30 + 0.05* % of body weight); in females it was considered the weight of the uterus and ovaries together. The gonads' weight reduction is an indication of the inhibition of physiologically produced sex hormones (Gusmão-Silva et al., 2022; Lichtenecker et al., 2021).

Table 1 shows the blood pressure values and renal function parameters. As expected, females treated with vehicle presented lower blood pressure values than males treated with vehicle. After hormonal treatment, the values obtained for the M + H group were also lower than those of the M + V group. On the other hand, the F + H group did not exhibit lower blood pressure values compared to M + V. The glomerular filtration rate, assessed by inulin clearance, did not show differences based on sex; however, hormonal treatment had a reducing effect, mainly in males (hormone effect: p = 0.0420). No differences were found between the groups in the values of renal plasma flow (PAH clearance). However filtration fraction was influenced by CHT. Urinary output was greater in females than in males. Hormonal treatment significantly increased urinary output in the M + H group. The excretion of titratable acids was greater in females. The ammonium excretion was similar in groups M + V and F + V; however, hormonal treatment reduced this excretion in males and increased in females. The arterial blood gas values (pH, pCO_2_, and HCO_3_^−^) did not show significant variations between the groups and were within the normal range. The glomerular area was expanded in males compared to females, and CHT did not change this parameter.

**TABLE 1:** Summary of blood pressure, glomerular area, and renal functional parameters in rats under cross-sex hormone therapy.

Parameter	M + V	M + H	F + V	F + H	Two-way-ANOVA		
	(N = 11)	(N = 10)	(N = 11)	(N = 7)	Interaction effect	Sex effect	Hormone effect
Blood Pressure (mmHg)	134.5 ± 2.5	124.8 ± 0.9*	120.7 ± 1.4^**#**^	126.6 ± 4.8	**p = 0.0029**	**p = 0.0195**	p = 0.4406
GFR – Inulin Clearance (mL/min/g kw)	0.64 ± 0.08	0.36 ± 0.04	0.69 ± 0.05	0.65 ± 0.11	p = 0.1469	**p = 0.0381**	**p = 0.0420**
RPF – PAH clearance (mL/min/g kw)	2.44 ± 0.35	2.17 ± 0.46	2.11 ± 0.14	3.02 ± 0.39	p = 0.1118	p = 0.4705	p = 0.3815
Filtration Fraction (%)	24.9 ± 3.7	18.4 ± 2.3	33.8 ± 2.0	22.1 ± 2.1*	p = 0.3208	**p = 0.0216**	**p = 0.0013**
Titratable acid excretion (µEq/min/g kw)	0.22 ± 0.03	0.19 ± 0.02	0.42 ± 0.06^**#**^	0.34 ± 0.05	p = 0.5623	**p = 0.0002**	p = 0.2009
Ammonium excretion (µEq/min/g kw)	0.34 ± 0.04	0.12 ± 0.02*	0.33 ± 0.06	0.36 ± 0.08^**#**^	**p = 0.0211**	**p = 0.0409**	p = 0.0942
Glomerular area mean (µm^2^)	8,597 ± 271	8,706 ± 385	7,070 ± 278^**#**^	7,730 ± 159	p = 0.3454	**p = 0.0003**	p = 0.1923
Urinary output (mL/24h/100 g)	3.21 ± 0.15	4.70 ± 0.23*	4.88 ± 0.25^**#**^	4.19 ± 0.43	**p = 0.0002**	**p = 0.0337**	p = 0.1377
Kidney weight (g)	3.37 ± 0.16	3.59 ± 0.17	2.62 ± 0.15^**#**^	2.57 ± 0.09^**#**^	p = 0.4121	**p < 0.0001**	p = 0.5955

Differences statistically significant when p < 0.05 (bold); vs. control* or male^**#**^. Tukey post test after two-way-ANOVA. Values are means ± standard error. The number of measurements is in parentheses.

**FIGURE 1:**
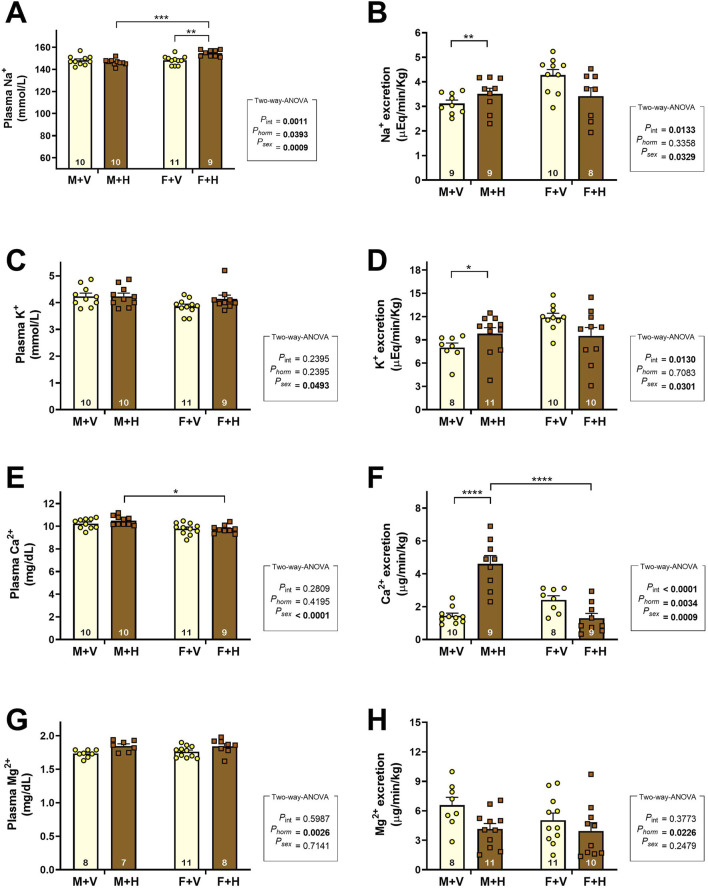
Plasma Concentrations and Urinary Excretion of Electrolytes in Rats Under Cross-Sex Hormone Therapy.Plasma concentrations **(A,C,E,G)** and urinary excretion (excreted load) **(B,D,F,H)** of sodium (Na^+^), potassium (K^+^), calcium (Ca^2+^), and magnesium (Mg^2+^) in 4-month-old Wistar rats subjected to cross-sex hormone therapy (CHT). Data are presented as mean ± standard error of the mean (SEM). The number of animals is shown inside the bars of the graphs. Statistical analysis was performed using two-way ANOVA. The p-values for hormone, sex, or interaction are shown on the right side of the graphs. Significant differences observed by Tukey’s post-test are marked with asterisks as follows: *p ≤ 0.05; **p ≤ 0.01; ****p ≤ 0.001.

The graphs presented in Figure 1 show the values found for plasma concentration and excreted load of Na^+^, K^+^, Ca^++^, and Mg^++^ ions. The CHT increased sodium plasma concentration in F + H (Figure 1A) and reduced sodium excretion in this group (Figure 1B/p_sex_ = 0.03; p_int_ = 0.0133) (Na^+^ excretion: M + V: 3.1 ± 0.14; M + H: 3.5 ± 0.23; F + V: 4.3 ± 0.22; F + H: 3.4 ± 0.34; μEq/min/Kg). Potassium excretion was higher in females than in males; CHT increased this excretion in the M + H group (Figure 1D/p_sex_ = 0.0301; p_int_ = 0.0130) (K^+^ excretion: M + V: 8.0 ± 0.57; M + H: 9.8 ± 0.77; F + V: 11.9 ± 0.52; F + H: 9.5 ± 1.09; μEq/min/Kg). Also in M + H group, CHT increased calcium excretion (Figure 1F/p_sex_ = 0.0009; p_horm_ = 0.0034; p_int_ = 0.0001) (Ca^++^ excretion: M + V: 1.4 ± 0.16; M + H: 4.6 ± 0.50; F + V: 2.4 ± 0.26; F + H: 1.3 ± 0.29; μEq/min/Kg) and reduced magnesium excretion (Figure 1H/p_horm_ = 0.0226) (Mg^++^ excretion: M + V: 6.6 ± 0.80; M + H: 4.1 ± 0.56; F + V: 5.0 ± 0.72; F + H: 3.9 ± 0.85; μEq/min/Kg).

**FIGURE 2:**
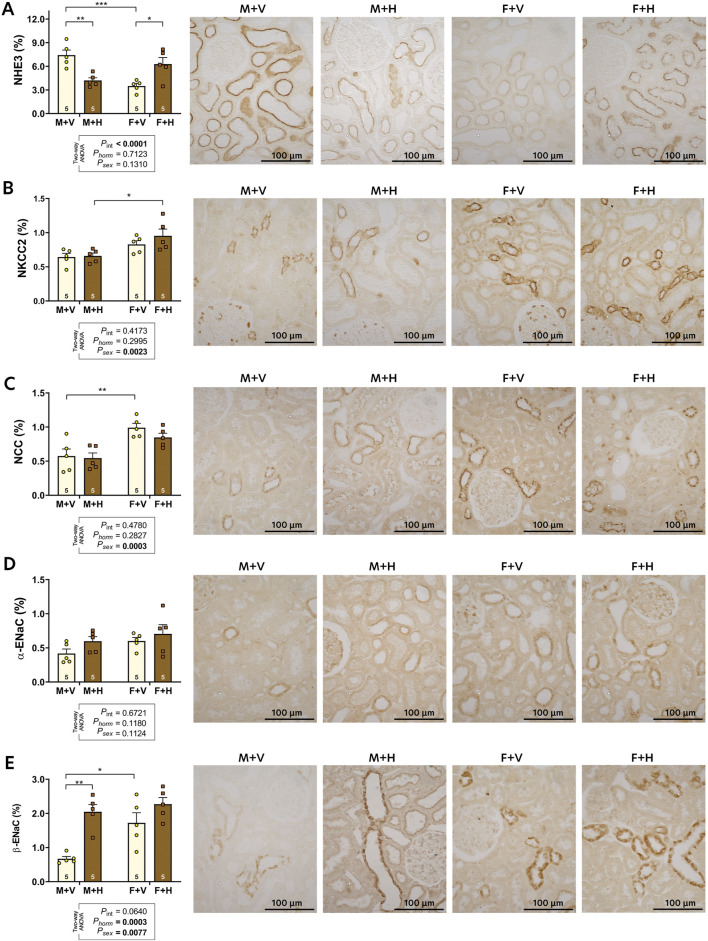
Renal expression of sodium transporters: NHE3 **(A)**, NKCC2 **(B)**, NCC **(C)**; and epithelial sodium channels: alpha-ENaC **(D)** and beta-ENaC **(E)** of 4-month-old rats submitted to CHT. Data are presented as mean ± standard error (SEM). Representative images of the immunohistochemical reaction for each transporter are shown beside the bar graph. The number of slides analyzed is shown within the bars of the graphs. Statistical analysis was performed using two-way ANOVA. The p-values for hormone, sex, or interaction are shown below the graphs. Significant differences observed by Tukey's post-test are marked with asterisks as follows: *p ≤ 0.05; **p ≤ 0.01; ****p ≤ 0.001.

The graphic representations of the expressions of sodium transporters (NHE3, NKCC, NCC) and epithelial sodium channels (ENaC) are presented in Figure 2. Increased expression of NHE3 was observed in M + V in comparison to F + V. The CHT decreased NHE3 expression in M + H and increased in F + H (Figure 2A). Sex differences were observed in NKCC expression (Figure 2B, p_sex_ = 0.0023), but CHT did not influence this result. The NCC expression was significantly higher in females than in males; CHT did not alter this pattern (Figure 2C). The αENaC expression was not different among the groups. However, βENaC was more expressed in females, and CHT in males (M + H) increased the expression of this protein (Figure 2E).

## Discussion

This study aimed to enlarge knowledge regarding renal adaptations resulting from cross-sex hormone therapy used in the gender identity-affirming process. To this end, we evaluated blood pressure, functional parameters, and the expression of tubular sodium transporters in male and female Wistar rats that were either subjected to or not subjected to this therapy.

Blood pressure evaluation is paramount, considering that hypertension is a common cardiovascular disorder and a risk factor for renal disease due to the progressive damage of glomerular capillaries (Carlström et al., 2015). BP alterations related to the use of sexual steroids have been the focus of several studies in experimental models and humans (cisgender situation) (ACOG, 2019; Reckelhoff, 2019; Tostes et al., 2003). In the present study, male rats under CHT (M + H) presented a significant reduction in BP, possibly related to the decrease in testosterone levels (Reckelhoff et al., 1998), alongside the increase in estradiol, known for its effects on smooth muscle, vascular, and endothelial cells (Tostes et al., 2003). Regarding the female groups, F + V, as expected, showed BP values smaller than M + V; however, F + H exhibited a slight increase in BP, which diminished the difference between this group and the M + V group. This effect may have occurred due to the rise in testosterone and/or reduction of estradiol blood levels in this group. Recently Banks et al. (2021) evaluated the BP from 470 transgender (247 trans-women and 223 trans-men) from a health center in Washington DC and observed that CHT significantly reduced the BP in trans-women; however, resulted in an increase in trans-men. The authors suggested that the hypotensive effect in trans-women could be both by the reduction of testosterone levels and by the use of spironolactone, also used in this population due to its anti-androgenic effects. Similar results were found by van Velzen et al. (2019) using cyproterone acetate in combination with estradiol valerate. Nevertheless, there are studies in which there was augmented BP in trans-women with different schedules of CHT (Colizzi et al., 2015; Elbers et al., 2003), highlighting the complexity of the subject.

Regarding the renal function parameters, no significant differences were observed between the control groups (M + V and F + V) in the clearances of the studied substances (inulin and PAH). However, in the M + H group, hormonal therapy caused a negative modulation in inulin clearance, suggesting a risk influence of the treatment on the glomerular filtration rate (GFR). In a large population-based cohort study, where cystatin clearance and plasma testosterone levels were assessed in men and women, a positive association was observed between testosterone and renal function in men. In contrast, in women, the association was negative (van der Burgh et al., 2023). The results of GFR obtained for the M + H group are consistent with this association, as the treatment reduces the natural production of testosterone. There were no alterations in renal plasma flow evaluated by PAH clearance.

Concerning urinary acid elimination, sexual dimorphism was observed in titratable acid excretion, where females excreted larger amounts compared to males. The titratable acid excretion is mainly composed of phosphate buffer. The larger part of the phosphate filtered is reabsorbed in the proximal tubule (PT), and only a small part is excreted. However, females have a lower proportion of phosphate transporters in PT, which may result in greater excretion of titratable acidity (Hu et al., 2019). Regarding ammonium ion excretion, there were no differences between the groups M + V and F + V; however, the CHT increased ammonium excretion in F + H and decreased in M + H. Harris et al. (2018) showed that testosterone exerts an important effect on ammonia’s metabolism in PT, possibly through androgenic receptors (AR), that stimulates phosphoenolpyruvate carboxykinase (PEPCK), an enzyme that is fundamental in ammonia genesis; in addition, is essential to emphasize that AR are expressed in PT of males and females. In this way, we may infer that the effects of CHT over ammonium excretion are possibly related to the alteration in testosterone levels in both M + H and F + H groups.

Dimorphism was also observed in urinary output, in which females (F + V) excreted a larger volume than males (M + V). Vasopressin action seems to be three times superior in males (due to testosterone) than in females (Wang et al., 1993), resulting in this difference. Furthermore, estradiol may reduce the antidiuretic effect of vasopressin, possibly through the estrogen receptor alpha (ERα) found in the collecting ducts of rats (Sladek and Somponpun, 2008). After CHT, male rats exhibited an increase in urinary output, probably due to the reduction in testosterone levels, in addition to the exogenous estradiol. The female group (F + H) did not show a significant decrease in this parameter.

Regarding sodium homeostasis, we observed that CHT caused an increase in plasma sodium concentration in females; this alteration is certainly related to the reduction of sodium excretion observed in this group. On the other hand, males under CHT exhibited an augment of urinary sodium excretion. These alterations reflect the renal adaptations resulting from a new hormonal pattern. To better understand these events, we evaluated the renal expression of sodium transporters from the experimental groups. The pattern of expression of sodium transporters in control rats (M + V and F + V) was similar to that observed by Veiras et al. (2017) in mice. These authors suggested that females have less developed PT, with fewer sodium transporters, mainly the sodium/hydrogen exchanger (NHE3). In addition, male hormones appear to exert an activating effect on NHE3 in PT, leading to higher sodium reabsorption in males (Quigley, 2008). CHT altered the expression of NHE3 in both sexes (M + H and F + H), possibly due to the changes in testosterone levels. In later segments of the nephron, sex differences were observed in the expression of sodium-chloride co-transporter (NCC) and the β-isoform of the epithelial sodium channel (β-ENaC). The NCC expression was higher in females than in males; the CHT did not change this feature. The β-EnaC expression was higher in females; however, CHT increased the expression in males. As proposed by McDonough et al. (2024), females at reproductive age, having a proportionally greater quantity of transporters in the distal nephron, can regulate sodium balance more effectively and excrete a saline overload more quickly than males, which makes them less susceptible to the development of arterial hypertension. In females under CHT, the expression of NHE3 increased significantly; however, in the distal nephron, there was no reduction in the expression of sodium transporters. These changes, taken together, may be responsible for the increase in plasma sodium concentration and reduced sodium excretion observed in F + H, which, with longer treatment, can lead to increased blood pressure, as previously observed (Lichtenecker et al., 2021).

Potassium excretion was significantly higher in females than in males. This result may be related to differences in urinary output between males and females. Although other factors may also modulate potassium excretion, it is well established that urinary flow can influence potassium secretion through BK channels (high-conductance channels activated by calcium) (Rieg et al., 2007). In the M + H group, besides the alterations in urinary output, potassium secretion may also have increased due to the augmented expression of ENaC.

Plasma concentrations of other vital electrolytes, such as calcium and magnesium, which are reabsorbed in the renal tubules, were also evaluated. CHT caused significant changes in calcium excretion. The PT is the nephron segment responsible for the major part of calcium reabsorption due to the formation of a favoring electrochemical trans-epithelial gradient that indirectly depends on the reabsorption of other solutes in this segment. Females, due to less developed PT, reabsorb less calcium in this segment compared to males. In the M + H group, the alterations caused by hormonal therapy may have influenced calcium reabsorption, resulting in increased excretion of this ion.

Regarding the renal balance of magnesium, M + H showed a reduction in the excretion of this ion. A significant portion of magnesium reabsorption occurs in the loop of Henle, in a way indirectly related to the activity of the NKCC co-transporter that is sensitive to vasopressin. As commented earlier, testosterone increases the activity of vasopressin (Wang et al., 1993); and its inhibition by CHT could decrease the activity of NKCC and indirectly reduce magnesium reabsorption. In another way, at late distal and collecting duct, magnesium reabsorption occurs through melastatin transient receptor potential cation channel (TRPM6 and TRPM7) (de Baaij, 2023); channels that seem to suffer hormonal regulation, including by estrogen that may increase the transcription and activity mainly of TRPM6 (Cao et al., 2009); through this effect, the magnesium excretion could have been reduced in M + H group.

Cross-sex hormone therapy plays a crucial role in affirming the identity of transgender individuals; however, its effects on renal function remain insufficiently understood. The present study shows that cross-sex hormone therapy in rats promotes adaptations in renal function in a sex-dependent way. CHT in females causes a reduction in sodium and other ion excretion, an alteration that, with prolonged treatment, may lead to an increase in blood pressure. Male rats under CHT presented a reduction in glomerular filtration rate, which may suggest inadequate renal adaptation. The expression of sodium transporters was altered uniquely, depending on sex and nephron segment.

Our findings provide valuable translational evidence indicating that cross-sex hormone therapy may lead to adaptive functional changes and alterations in blood pressure. However, the values of this parameter remained within the normal range. These results enhance our understanding of potential risks that warrant monitoring in transgender individuals receiving cross-sex hormone therapy.

Although experimental results cannot be directly extrapolated to humans, such data are crucial for understanding physiological changes under various conditions. Moreover, research using animal models offers valuable insights into how hormone treatments, such as those employed in gender affirming hormone therapy, influence bodily functions and health parameters. These studies are essential for assessing potential health risks, advancing clinical research, and addressing gaps in our current knowledge regarding the outcomes of hormone therapy (Tammaro et al., 2024). Additional studies are still needed to specifically appreciate the repercussion of CHT on renal function and the consequences of using this therapy for a longer time.

## Data Availability

The raw data supporting the conclusions of this article will be made available by the authors, without undue reservation.
